# Inflammatory markers and frailty in home-dwelling elderly, a cross-sectional study

**DOI:** 10.1186/s12877-024-04690-2

**Published:** 2024-02-19

**Authors:** Pia Bålsrud, Stine M. Ulven, Jacob J. Christensen, Inger Ottestad, Kirsten B. Holven

**Affiliations:** 1 Department of Nutrition, Institute of Basic Medical Sciences, University of Oslo, Oslo, Norway; 2 National Advisory Unit on FH, Oslo University Hospital, Oslo, Norway; 3 Clinical Nutrition, Department of Clinical Service, Division of Cancer Medicine, Oslo University Hospital, Oslo, Norway

**Keywords:** Frailty, Frailty Index, Inflammation, Ageing, Inflammageing, Home-dwelling, Cross-sectional, Gene expression, PBMC

## Abstract

**Background:**

Low-grade, chronic inflammation during ageing, (“inflammageing”), is suggested to be involved in the development of frailty in older age. However, studies on the association between frailty, using the frailty index definition, and inflammatory markers are limited. The aim of this study was to investigate the relationship between inflammatory markers and frailty index (FI) in older, home-dwelling adults.

**Method:**

Home-dwelling men and women aged ≥ 70 years old, living in South-East Norway were recruited and included in a cross-sectional study. The FI used in the current study was developed according to Rockwood’s frailty index and included 38 variables, resulting in an FI score between 0 and 1 for each participant. Circulating inflammatory markers (IL-6, CRP, IGF-1, cystatin C, cathepsin S, and glycoprotein Acetyls) were analyzed from non-fasting blood samples using ELISA. Whole-genome PBMC transcriptomics was used to study the association between FI score and inflammation.

**Results:**

The study population comprised 403 elderly (52% women), with a median age of 74 years and a mean BMI of 26.2 kg/m^2^. The mean FI score for the total group was 0.15 (range 0.005–0.56). The group was divided into a frail group (FI score ≥ 0.25) and non-frail group. After adjusting for BMI, age, sex, and smoking in the whole group, IL-6, cathepsin S, cystatin C, and Gp-acetyls remained significant associated to FI score (IL-6: 0.002, 95% CI: 0.001, 0.002, cathepsin S: 6.7e-06, 95% CI 2.44e-06, 0.00001, cystatin C: 0.004, 95% CI: 0.002, 0.006, Gp- Acetyls: 0.09, 95% CI: 0.05, 0.13, *p* < 0.01 for all), while CRP and IGF-1 were not (0.0003, 95% CI: -00001, 0.0007, *p* = 0.13, (-1.27e-06), 95% CI: (-0.0003), 0.0003, *p* = 0.99). There was a significant association between FI score and inflammatory markers, and FI score and monocyte-specific gene expression.

**Conclusions:**

We found an association between FI score and inflammatory markers, and between FI score and monocyte-specific gene expression among elderly subjects above 70 years of age. Whether inflammation is a cause or consequence of frailty and whether the progression of frailty can be attenuated by reducing inflammation remains to be clarified.

**Supplementary Information:**

The online version contains supplementary material available at 10.1186/s12877-024-04690-2.

## Introduction

The number of people aged 60 years and older is increasing worldwide [1]. This emphasizes the importance of maintaining good health and the ability to live longer at home for older individuals. It not only improves their quality of life but also prevents the burden of poor health on society [1].

Frailty is a condition associated with increased vulnerability to adverse health outcomes [2]. It reflects multisystem physiological changes and is commonly defined by a frailty index (FI), described by Rockwood et al. [3]. A FI considers signs, symptoms, disabilities, diseases, and laboratory measurements, termed “deficits”. The number of deficits refers to the degree of frailty [3].

Low-grade, chronic inflammation during ageing, known as “inflammageing”, is suggested to contribute to the development of frailty in older age [4, 5]. There is increasing evidence that elevated levels of inflammatory markers are associated with phenotypes of ageing [4], potential drivers and mechanisms include central obesity, cellular senescence, genetic susceptibility, activation of the inflammasome and dysregulation of inflammatory cells, oxidative stress, microbiota composition, gut permeability, and chronic infections [6]. Also, higher circulating levels of inflammatory markers are associated with loss of muscle mass and strength, as well as cognitive decline in older adults [6, 7].

Inflammation is involved in cardiovascular disease (CVD), multimorbidity, and frailty by inhibiting growth factors, increasing catabolism, and is considered as a significant factor in the biology of ageing [6, 8]. However, an important question is whether inflammation is the cause of the pathology or only a biomarker of the rate of biological ageing [6].

To the best of our knowledge, only a few studies have examined the association between frailty, using the FI definition, and inflammatory markers. One study on subjects aged 65–75 years at the study endpoint showed significant associations between low-grade inflammation and FI [9]. Another study showed an association between CRP trajectories over a follow-up time of more than 15 years and FI, in subjects aged 60–85 at endpoint [10].

To further explore the association between frailty and inflammation, the aim of the present study was to investigate the association between frailty using the FI, and plasma inflammatory biomarkers (IL-6, CRP, IGF-1, cystatin C, cathepsin S, Gp-acetyls) and inflammatory gene expression in peripheral blood mononuclear cells (PBMCs) among home-dwelling elderly people (≥ 70 years).

## Method

### Study population and design

The present study was a cross-sectional study that included home-dwelling men and women aged ≥ 70 years old, living in the Skedsmo area, South-East Norway. The study was conducted in 2014/2015 and has been described previously [11]. The participants were recruited by the National Register and received an invitation letter by mail. Briefly, a total of 2820 subjects were invited, and 437 subjects participated in the study. The participants met for a single study visit, and data was collected on dietary intake, body weight and composition, physical performance, medical history, cognitive function, risk of malnutrition, anthropometric measurements, blood pressure, heart rate, and quality of life. Non-fasting blood samples were also collected.

This study was conducted according to the guidelines in the Declaration of Helsinki and approved by the Regional Committees for Medical and Health Research Ethics, Health Region South East, Norway (2014/150/REC). Written informed consent was obtained from the participants.

### Frailty index

FI was constructed following the procedure described by Searle et al. [2]. Additional studies were used to supplement the establishment of the FI [12–14]. A flowchart describing the process of creating the FI is shown in Supplementary Fig. 1. Briefly, potential health-related variables collected in our study were evaluated based on the following 5 criteria described by Searle et al. [2]: The variables must be deficits associated with health, they should generally increase with age, they should not saturate too early (as for example age-related lens changes do), they should cover a range of systems, and if used serially on the same people, they should be consistent across time points. To generate the index, at least 30–40 health deficits should be used. Each deficit is represented by either a binary variable, taking the values 0 or 1, or as an ordinal variable from 0 to 1 (grading). The absence of the deficit is represented by “0”, while “1” indicates the presence of a deficit. The index is then calculated by taking the sum across deficits and dividing the sum by the total number of deficits [2]. The FI is then presented as a proportion that can be examined, as a continuous variable.

**Fig. 1:**
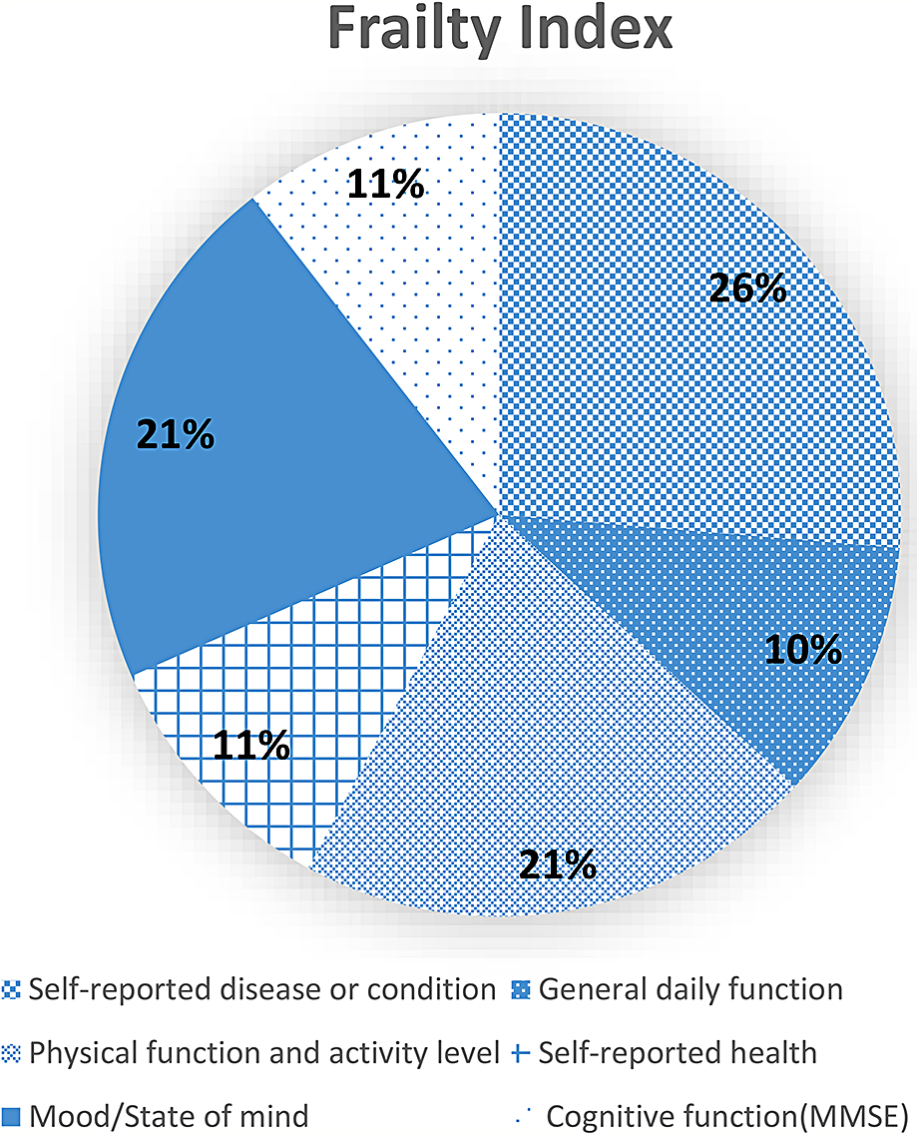
The composition of the frailty index. The pie chart shows the sub-headings to categorize the 38 deficits included in the fraity index. An even distribution of deficits to each category will ensure that the index cover a range of systems

For the present work the FI was constructed based on 38 deficits categorized into the subheadings “Self-reported disease or condition”, “General daily function”, “Physical function and activity level”, “Self-reported health”, “Mood/State of mind”, and “Cognitive function (MMSE)” (Fig. 1). See Supplementary table 2a for the list of deficits that were included to create the FI.

Missing variables in ≥ 20% of the population were excluded from the analyses (*N* = 5). For participants with missing data < 20%, the number of deficits present was divided by the total number of deficits measured. For example: if a subject had a single missing variable, the numerator of the ratio was 38 − 1 = 37 [15].

The FI was primarily used as a continuous variable. Also, we divided the participants into a frail and a non-frail group, where the frail group was defined as an FI score ≥ 0.25, while the non-frail group was defined as an FI score < 0.25. This categorization of frailty in home-dwelling elderly people is in agreement with Rockwood et al. [16] and used in previous studies to define a frailty cut-off [17–20]. Both women and men were investigated in the total study sample, as well as in the frail and non-frail subgroups.

### Circulating biomarkers

Non-fasting venous blood samples were collected by trained bioengineers at the study visit, as previously described [11]. Briefly, serum samples were centrifuged and then either frozen at -80 °C or used immediately for routine measurements (TC, TG, LDL-C, HDL-C, CRP) at FÜRST Medical Laboratory (Oslo, Norway). Whole blood was collected into EDTA tubes, and centrifuged to obtain plasma which was stored at -80 °C. Whole blood was used immediately for analysis of hemoglobin and HbA1c, using standard methods. IL-6, IGF-1, cystatin C, and cathepsin S were measured using Enzyme-Linked Immunosorbent Assay (ELISA) at the University of Oslo, Norway. Gp-acetyls were measured as a part of a comprehensive metabolomics assessment using nuclear magnetic resonance (NMR) spectroscopy (Nightingale, Finland).

### PBMC gene expression

Blood samples were collected in non-fasting state in BD Vacutainer® CPT™ cell preparation tubes with sodium heparin (Becton Deckenson, NJ, USA). PBMCs were stored at − 80 °C until mRNA was extracted using RNeasy Mini Kit (Qiagen), as described elsewhere [21]. RNA quantity was measured using NanoDrop-1000 (NanoDrop Technologies, Inc., Delaware, USA), while RNA quality was checked with Aglient 2100 Bioanalyzer (Agilent Technologies, Inc., California, USA).

After RNA preparation and amplification, using the Illumina Total Prep RNA Amplification Kit (Illumina Inc., California, USA), gene expression measurements were performed by hybridizing the amplified RNA to Illumina HumanHT-12 v4 Expression BeadChip (Illumina Inc., California, USA) according to the manufacturer’s instructions. This provided genome-wide measurements of the expression of 11, 925 genes with over 48,000 probe sets, as previously described [22]. One probe per gene (max IQR) was selected for further analysis. The microarray experiments were conducted according to the MIAME (Minimum Information about a Microarray Experiment) guidelines.

After correcting for background noise, using normexp background correction (neqc filtration, Limma), quantile normalization of the data was performed using the Illumina GenomeStudio software, version 1.7.0. Data were log2-transformed and exported raw (non-normalized) to R [23] for biostatistical analysis.

### Monocyte-specific gene expression and FI

Because PBMCs comprise a heterogeneous pool of leukocytes, associations between gene expression and FI score could be confounded by cell type. A high number of tests also increases the probability of false positive findings. Therefore, we subjected the entire PBMC gene expression matrix to CIBERSORT analysis, which is an in silicio flow cytometry cell type quantification. The association between CIBERSORT-predicted monocytes (as a relative proportion of entire PBMC pool) and FI score was analyses by linear regression models (both unadjusted and adjusted for age and BMI (Fig. 4)). Further, we explored the correlation between CIBERSORT-predicted monocytes and monocyte-related genes, and used the 30 most significant correlated genes in further analysis. The association between inflammatory markers (IL-6, CRP, Gp-acetyls, IGF-1, cystatin C and cathepsin S) and top 30 monocyte-specific genes was analyzed by linear regression both unadjusted and adjusted for BMI and age (Fig. 5).

### Statistical analyses

Normally distributed data were presented as mean ± standard deviation (SD), while non-normally distributed data were presented as median (min-max). Categorical variables were presented as frequencies (n) and relational proportions (%). For continuous variables, independent sample t-test and Mann-Whitney U test were used on normal distributed and non-normally distributed data, respectively. The chi-square test was used for categorical data, while Fisher’s exact test was used for small groups.

Non-normally distributed data were log-transformed (CRP, IL-6, and cystatin C), and Spearman’s correlation was used for the correlation analyses. Linear regression analyses were used to examine the association between inflammatory markers (independent) and FI score (dependent). Adjustment variables were age, sex, BMI, and smoking. The log-transformed β coefficients, and 95% CIs were back-transformed (variable multiplied with log(1.1)), which gave us the change in FI score with a 10% increase in the variables IL-6, CRP, and cystatin C.

For the PBMC gene expression analyses, in an initial untargeted approach, we fitted standard linear regression models for each of the 11 925 mRNA transcripts with FI score as the main exposure. The main model was adjusted for age and BMI; we visualized the regression coefficients and *P* values in a Volcano plot. Two other models were fitted: a crude, unadjusted model, and a model adjusted for age, BMI, and proportion of monocytes and lymphocytes in blood (not shown).

In a more targeted approach, we sought to explore cell type-specific genes. To derive an estimate of cell type proportions in the PBMC pool, we performed gene expression decomposition (*in silico* flow cytometry) using CIBERSORT [24]. We fitted regression models for each CIBERSORT-predicted cell type with FI score as the main exposure (both crude models and models adjusted for age and BMI). We then extracted the top 30 genes/mRNA transcripts associated with CIBERSORT-predicted monocytes and associated them with the six serum inflammatory biomarkers in linear regression models adjusting for age and BMI. The resulting β coefficient matrix was visualized as a heatmap (ComplexHeatmap R package) (Fig. 5).

The level of significance was defined as *P* < 0.05, and all tests were two-sided. Analyses were performed using STATA Windows (version 17.0) and R (version 4.2.3) using the RStudio IDE [23].

## Results

### Study population

Four hundred and three participants were included, of which 210 (52%) were women (Table 1). The median age was 74 years (min-max 70–93), the mean BMI was 26.2 kg/m^2^ (SD ± 3.9) and 34% were living alone. Seventy subjects were categorized as frail (17% FI score ≥ 0.25), while 333 (83%) were categorized as non-frail (FI score < 0.25) [16]. Within the frail group, 40 (57%) were women and 30 (43%) were men. Compared to the non-frail subjects, the frail subjects had higher median age, a higher proportion were living alone, and they had lower mean levels of total cholesterol and LDL-C (Table 1). Among women, the median age was the only variable that was significantly different between frail and non-frail subjects (Supplementary Table 1). Among men, frail subjects were older, and a higher proportion were living alone (Supplementary Table 1).

**Table 1:** Descriptive data of the study population

Variable	Total (*N* = 403)	Frail (*N* = 70)	Non-frail (*N* = 333)	*P*-value
Age, years	74 (70–93)	78 (70–90)	74 (70–93)	**< 0.001**
Female, *n* (%)	210 (52)	40 (57)	170 (51)	0.35
BMI, kg/m^2^	26.2 ± 3.9	26.8 ± 4.4	26.0 ± 3.8	0.13
Daily smoking, *n* (%)	24 (6)	3 (4)	21 (6)	0.78
Living alone, *n* (%)	137 (34)	34 (49)	103 (31)	**0.005**
FI score	0.15 (0.005–0.56)	0.31 (0.25–0.56)	0.13 ± 0.06	**< 0.001**
*Blood markers*				
TG, mmol/L	1.33 (0.5-5.0)	1.24 (0.5–4.1)	1.36 (0.5-5.0)	0.30
Total Cholesterol, mmol/L	5.3 ± 1.1	5.1 ± 1.1	5.3 ± 1.1	***0.05***
LDL-cholesterol, mmol/L	3.1 ± 1.0	2.9 ± 1.0	3.2 ± 0.9	**0.03**
HDL-cholesterol, mmol/L	1.6 ± 0.5	1.6 ± 0.55	1.6 ± 0.49	0.83
HbA1c, %	5.9 ± 0.7	5.9 ± 0.5	5.9 ± 0.7	0.80
Glucose, mmol/L	4.4 (2.96–20.7)	4.4 (3.2–9.7)	4.4 (2.96–20.7)	0.75

*Cut-off value to be categorized as “frail” was set to ≥ 0.25. normal distributed data is presented as mean ± SD, non-normally distributed data is presented as median (min-max).P-values: Continuous normal distributed data were tested by t-test, continuous non-normally distributed data were tested by Mann-Whitney U test. Chi-square test was used for categorical data*. *Statistical significant level:P-value < 0.05. BMI, body mass index; FI score, Frailty Index score; LDL, low-density lipoprotein; HDL, high-density lipoprotein*

### Inflammatory markers

In the whole population, there was a significant positive correlation between FI score and CRP (R: 0.20, *p* < 0.001), IL-6 (R: 0.28, *p* < 0.001), cathepsin S (R: 0.17, *p* < 0.001), cystatin C (R: 0.28, *p* < 0.001), and Gp-acetyls (R: 0.19, *p* < 0.001) as shown in Table 2. Among women, a positive correlation was found between FI score and CRP (R: 0.18, *p* = 0.01), IL-6 (R: 0.27, *p* < 0.001), cystatin C (R: 0.28, *p* < 0.001), cathepsin S (R: 0.21, *p* = 0.003), and Gp-acetyls (R: 0.17, *p* = 0.014). For men, we found a significant positive correlation between FI score and CRP (R: 0.22, *p* = 0.002), IL-6 (R: 0.33, *p* < 0.001), cystatin C (R: 0.33, *p* < 0.001), cathepsin S (R: 0.16, *p* = 0.03), and Gp-acetyls (R: 0.21, *p* = 0.004).

**Table 2:** Spearman‘s correlation between FI score and inflammatory markers

Inflammatory marker	Total (*N* = 403)		Women (*N* = 210)		Men (*N* = 193)	
	***R***	***P-value***	***R***	***P-value***	***R***	***P-value***
CRP** (*N* = 400), mg/L	0.20	**< 0.001**	0.18	**0.01**	0.22	**0.002**
IL-6* (*N* = 394), pg/ml	0.28	**< 0.001**	0.27	**< 0.001**	0.33	**< 0.001**
IGF-1, ng/ml	-0.1	0.04	-0.1	0.16	-0.08	0.26
Cystatin C*, ng/ml	0.28	**< 0.001**	0.28	**< 0.001**	0.33	**< 0.001**
Cathepsin S, pg/ml	0.17	**< 0.001**	0.21	**0.003**	0.16	**0.03**
Gp-acetyls, mmol/L	0.19	**< 0.001**	0.17	**0.014**	0.21	**0.004**

Variables with “*” are log-transformed before the correlation. Spearman’s correlation is used in all of the correlation analyses. ** CRP < 50: CRP levels ≥ 50 were excluded from the analysis and log-transformed. IL-6; interleukin-6, IGF-1; insulin-like growth factor-1, CRP; C-reactive protein, Gp-acetyls; Glycoprotein acetyls

**Fig. 2:**
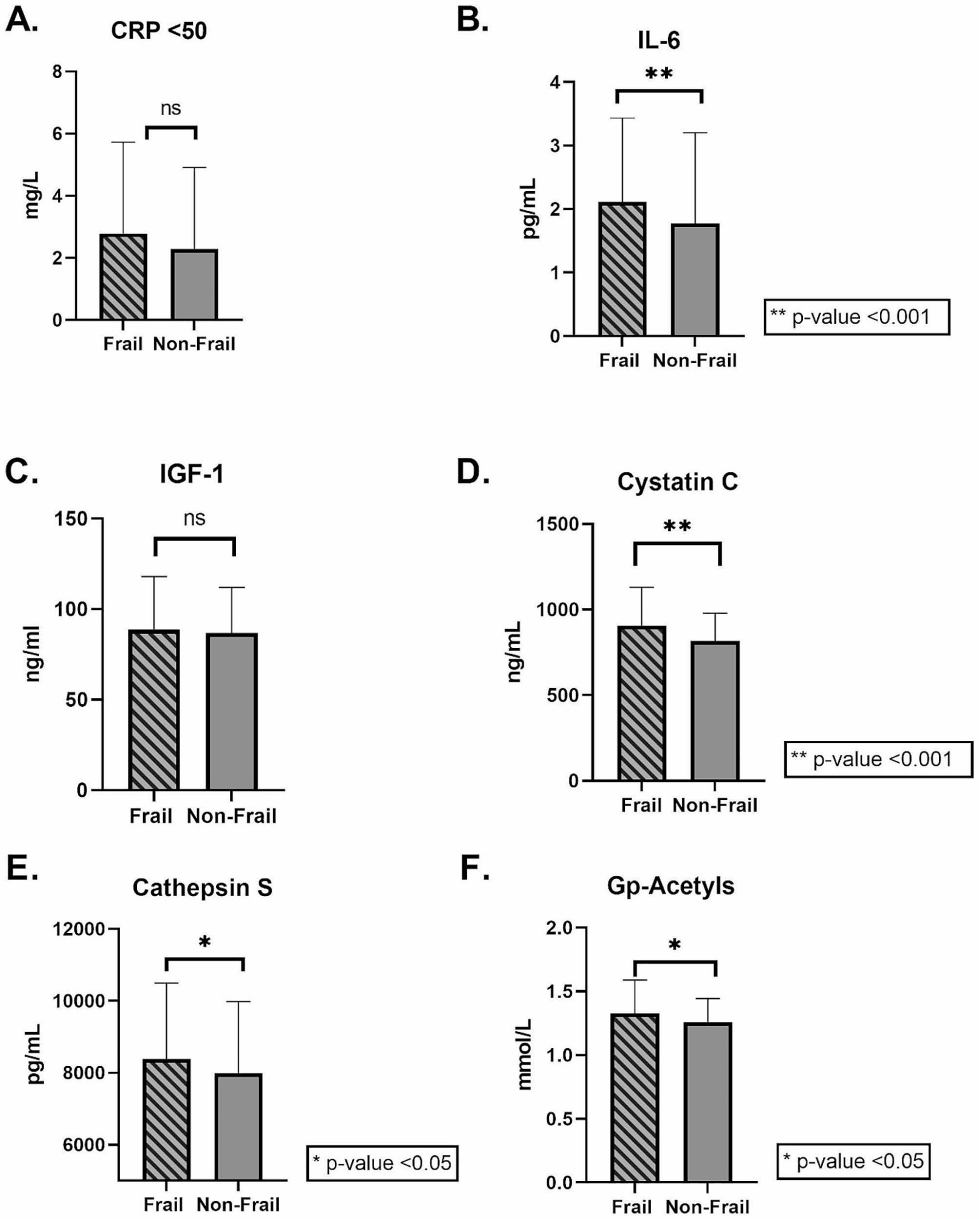
Inflammatory markers in frail vs. non-frail group. The bar charts show the differences in the concentration of inflammatory markers in the frailty groups, for; A.) CRP, B.) IL-6, C.) IGF-1, D.) cystatin C, E.) cathepsin S, and F.) Gp-acetyls. The data are an illustration of supplementary Table 3a and show significantly higher levels of IL-6 cystatin C, cathepsin S, and Gp-acetyls in the frail group, compared to the non-frail group. There were no significant differences in CRP or IGF-1 between the two groups. The cut-off value to be categorized as “frail” was set to ≥ 0.25. CRP levels ≥ 50 were excluded from the analysis. * P<0.05, **P<0.001. CRP, C-reactive protein; IL-6, interleukin 6; IGF-1, insulin growth factor 1; Gp-acetyls, Glycoprotein acetyls

The levels of IL-6 (1.77 pg/ml (0.47-8.9) vs. 1.33 pg/ml (0.37-10.0)), cystatin C ng/ml(852.9 (551.2-1864.6) vs. 778.9 ng/ml (496.9-1871.1)), cathepsin S (8384.8 pg/ml ± 2114.0 vs. 7980.9 pg/ml ± 1996.5), and Gp-acetyls (1.33 mmol/L ± 0.26 vs. 1.26 mmol/L ± 0.18) were significantly higher in the frail group compared to the non-frail group (Fig. 2 and Supplementary Table 3a), *p* < 0.05 for all. Only the level of cystatin C was significantly different between the frail women compared to frail men (827.5 ng/ml (551.3-1435.5) vs. 931.9 ng/ml (664.4-1864.6)*, p* = 0.03). Whereas non-frail women had significantly lower levels of IGF-1 (84.0 ng/ml ± 23.39 vs. 89.76 ng/ml ± 26.47) and cystatin C (764.7 ng/ml (538.1-1871.1) vs. 806.6 ng/ml (496.9-1403.4) compared to non-frail men, *p* < 0.05. Frail women had significantly higher levels of IL-6 (1.63 pg/ml (0.47–5.85) vs. 1.3 pg/ml (0.45-10.0)) and cystatin C (827.5 ng/ml (551.3-1435.5) vs. 764.7 ng/ml (538.1-1871.1)) compared to non-frail women, *p* < 0.05. Frail men had significantly higher levels of IL-6 and cystatin C compared to non-frail men. The levels of CRP (2.5 mg/L (0.4–17) vs. 1.3 mg/L (0.2–23), *p* = 0.05) tended to be higher among frail men when compared to non-frail men (Supplementary Table 3b for all above).

### Multiple regression model

After adjusting for BMI, age, sex, and smoking, CRP was not significantly associated with FI score anymore (0.0003, 95% CI: -0.0001, 0.0007, *p* = 0.13), while IL-6, cathepsin S, cystatin C, and Gp-acetyls remained significant (IL-6: 0.002, 95% CI: 0.001, 0.002, cathepsin S: 6.7e-06, 95% CI 2.44e-06, 0.00001, cystatin C: 0.004, 95% CI: 0.002, 0.006, Gp-acetyls: 0.09, 95% CI: 0.05, 0.13, *p* < 0.01 for all) (Supplementary Table 4).

### Gene expression in PBMC

In a subgroup of the participants, PBMC whole genome expression data was available (*n* = 89). In this subgroup, only women were included, the mean age was 78.1 years (SD ± 5.2), the mean BMI was 25.5 kg/m^2^ (SD ± 4.1), and 47 (53%) were living alone (Supplementary Table 5). Correlation analyses between inflammatory markers and FI score in the subpopulation are shown in Supplementary Fig. 2.

**Fig. 3:**
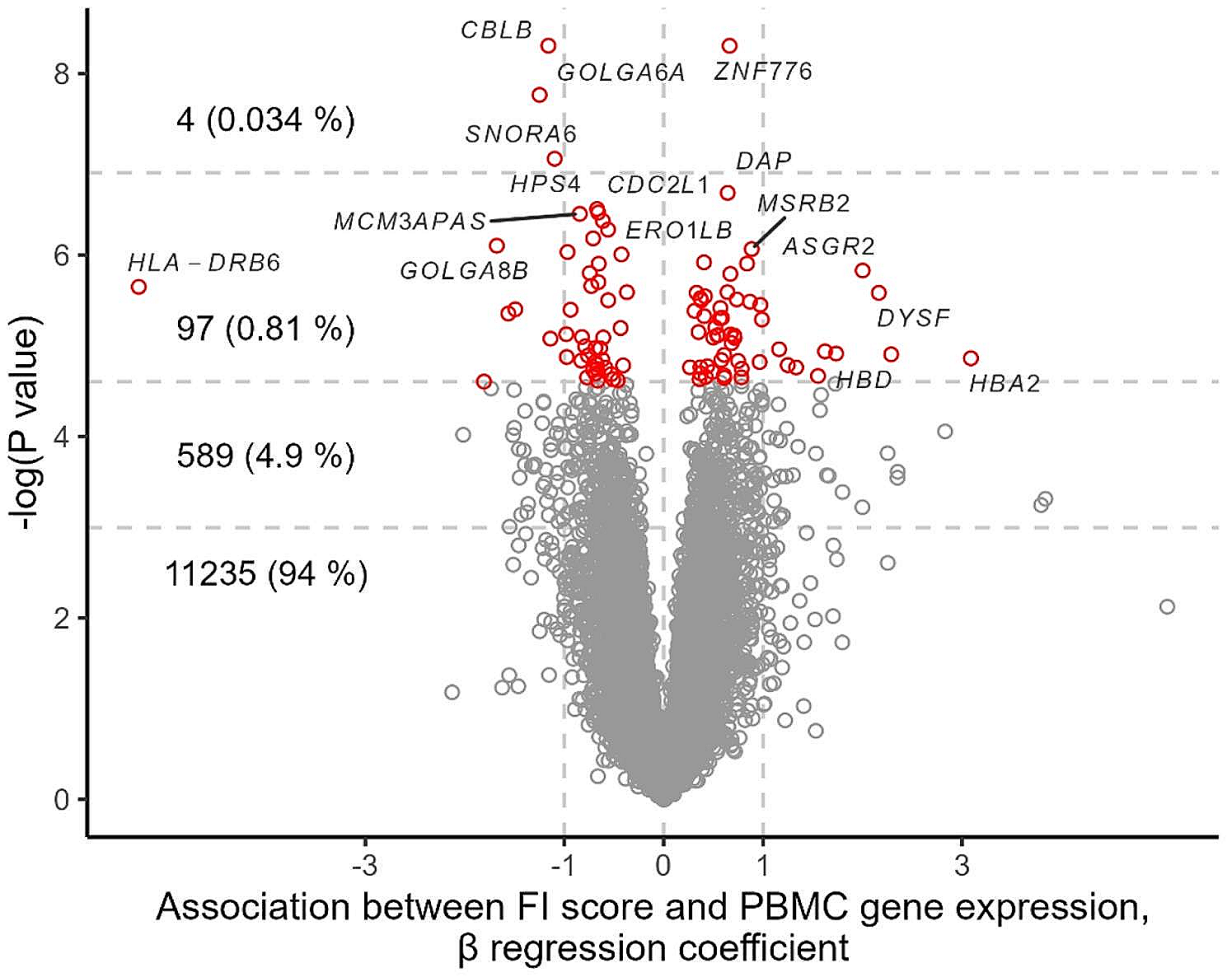
Association between frailty index score and PBMC gene expression. The volcano plot shows the β regression coefficient (x-axis) and–log *P-*value (y-axis) for each gene transcript (total number of 11 925 transcripts). Horizontal lines correspond to (from the bottom) *P-*value thresholds 0.05, 0.01, and 0.001; the labels display the counts and proportions of the total number of transcripts within each *P-*value category. The annotated genes are a random subset among genes with *P* < 0.01. Abbreviations: FI, frailty index; PBMC, peripheral blood mononuclear cells

**Fig. 4:**
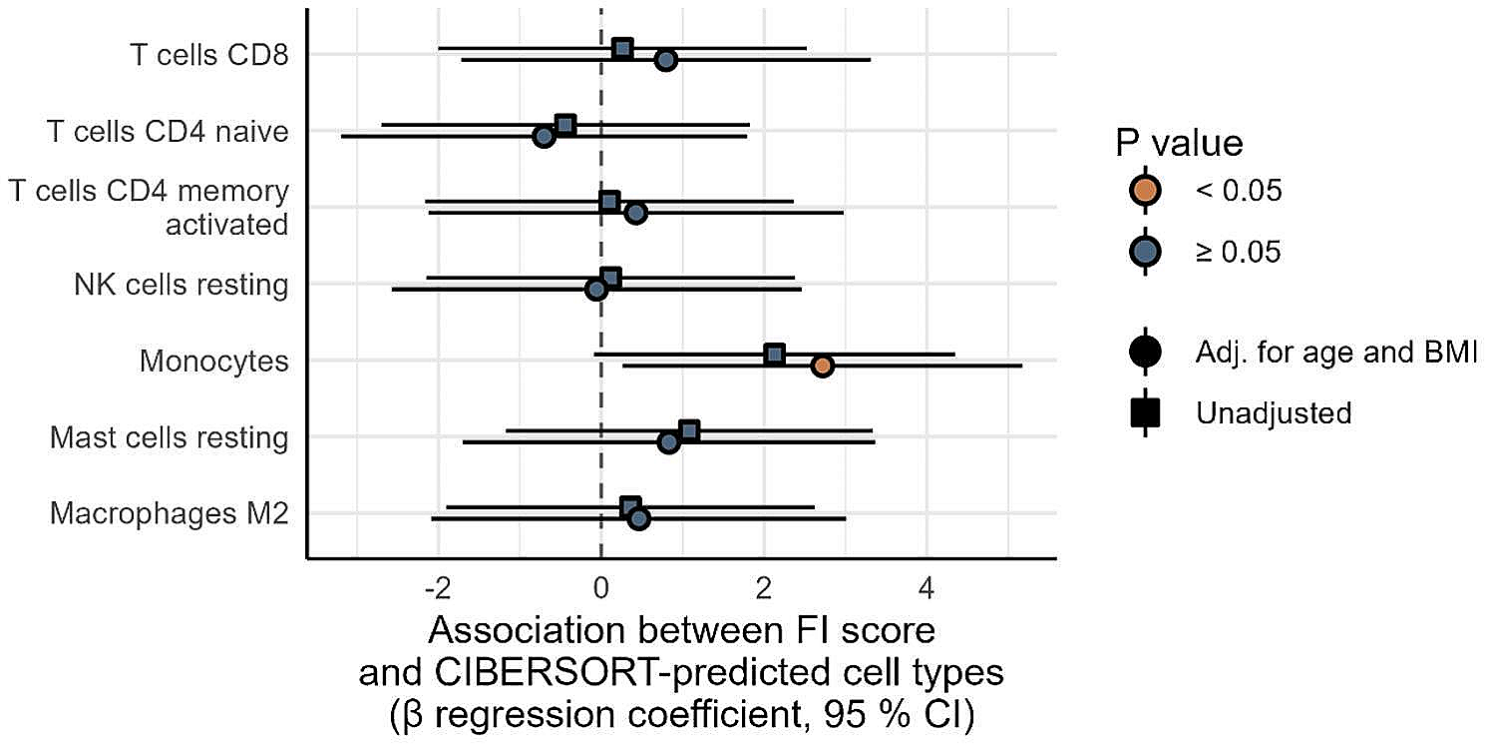
Association between Frailty index score and CIBERSORT-predicted cell types. The forest plot shows the unadjusted and adjusted (for age and BMI) β regression coefficients with 95% confidence intervals (CIs), colored by *P-*value. Abbreviations: CD4, cluster of differentiation 4; CD8, cluster of differentiation 8; FI, frailty index; NK, natural killer

To explore the association between FI score and inflammatory gene expression, we performed whole-genome transcriptomics in PBMC. A total of 11 925 genes were expressed in the PBMC, of which 589 (4.9%), 97 (0.81%), and 4 (0.034%) were significant associated with the FI score, with *P*-values of 0.05, 0.01, and 0.001, respectively. However, after adjustment for multiple testing, no genes were significantly associated with the FI score. (Fig. 3). We then examined cell types in the PBMC pool using gene expression decomposition. FI score associated with CIBERSORT-predicted monocytes (Fig. 4), likely representing a monocyte-specific gene expression signal independent of the most nominally significant genes.

**Fig. 5:**
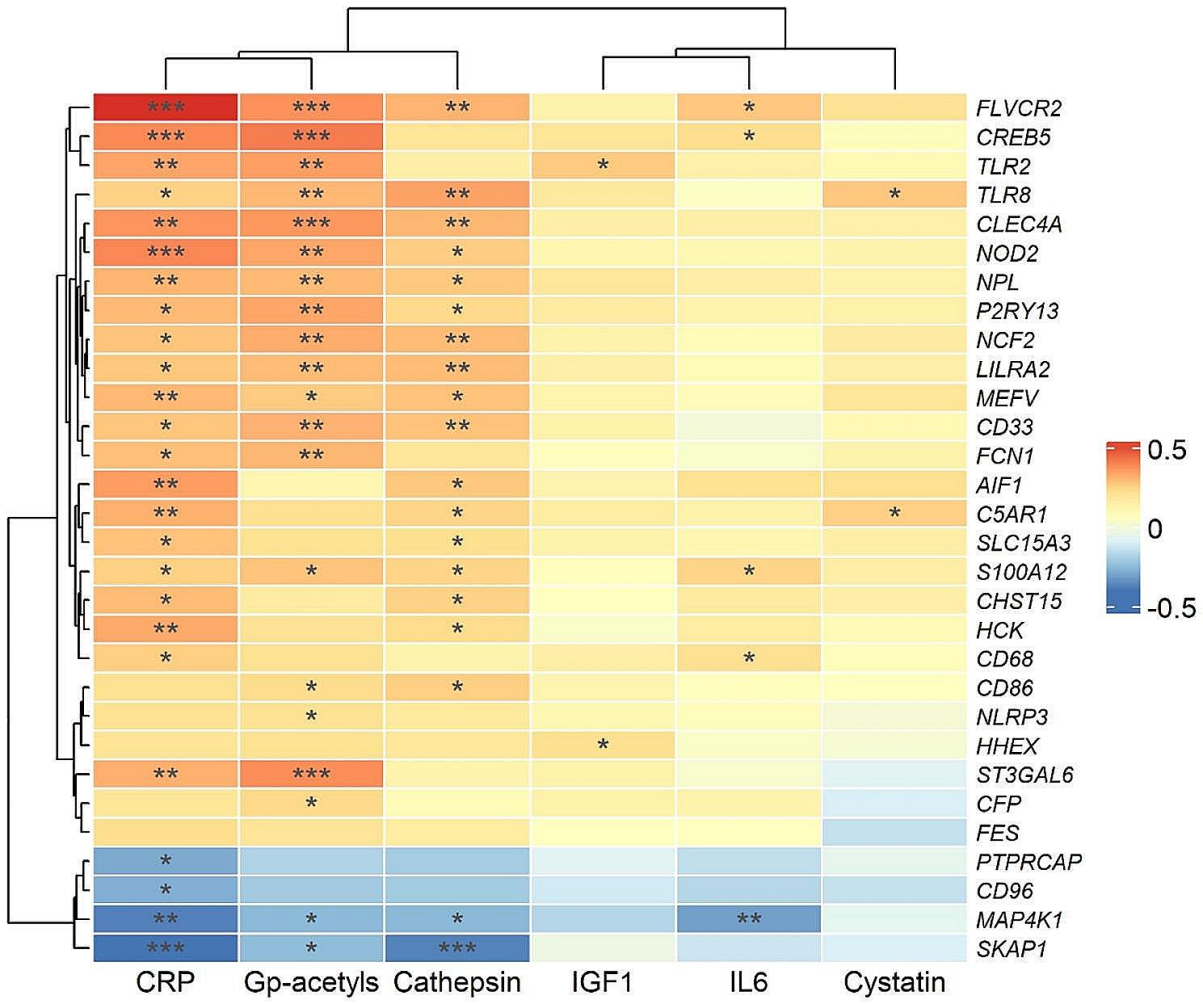
Association between serum inflammatory biomarkers and monocyte-specific genes. The heatmap shows β regression coefficients between serum inflammatory biomarkers and PBMC gene expression for the top 30 monocyte-specific genes, as derived from a CIBERSORT analysis. The models were adjusted for age and BMI; to aid comparison, we scaled both inflammatory biomarkers and genes to a standard normal distribution (with mean zero and standard deviation one). Rows and columns are clustered by hierarchical clustering (Euclidean distance). Abbreviations: CRP, C-reactive protein; IGF1, insulin-like growth factor-1; IL6, interleukin 6; Gp-acetyls, glycoprotein acetyls. * *P* < 0.05, ** *P* < 0.01, *** *P* < 0.001

Finally, we extracted the top 30 genes most significantly related to CIBERSORT-predicted monocytes (including typical monocyte marker genes like NOD2, NLRP3, CD68, and several TLRs), and explored their association with the serum inflammatory biomarkers. As expected, the associations were weak to moderate (absolute values for standardized β-coefficients mostly in the range of 0.05–0.35, Fig. 5). In general, however, the associations were stronger for CRP, Gp-acetyls, and cathepsin, indicating a more robust signal for these biomarkers.

### Sex differences

Frail women had higher levels of total cholesterol and HDL-C compared to frail men (Supplementary Table 1). More of the non-frail women were living alone, had significant higher levels of total cholesterol, LDL-C, and HDL-C, than non-frail men (Supplementary Table 1).

Some of the frailty variables that made up the FI are presented in Supplementary Table 2a and 2b. Compared to women, a significantly higher proportion of men had cancer and CVD, and lower blood levels of hemoglobin. Men had higher daily use of medications on prescription; however, they also reported to feel a higher level of energy in life, and showed higher grip strength, compared to women. Compared to men, more women used TSH medications, and showed greater limitations in self-reported performance in moderate activities of daily living, as well as limitations to lift/carry a shopping basket. (Supplementary Table 2b).

Frail men had more often cancer, and used more medications, compared to frail women. Whereas more frail women reported difficulties to lift/ carry a shopping basket compared to frail men.

## Discussion

In this study of home-dwelling elderly, we found that several inflammatory markers were associated with frailty. The FI showed positive associations with CRP, IL-6, cathepsin S, cystatin C, and Gp-acetyls in both sexes. The FI score was also associated with monocyte-specific gene expression related to inflammation. While the link between inflammation and frailty is evident, whether inflammation is a cause or consequence of frailty remains uncertain.

Previous studies have suggested that markers like IL-6 and CRP play a role in the transition to frailty [25, 26], while Gp-acetyls and cathepsin S has been linked to mortality risk [27–29]. Our study, being the first to investigate Gp-acetyls and cathepsin S, and frailty, highlights their potential as markers for frailty. Gp-acetyls are acute-phase proteins, which increase during inflammation and are used as a clinical marker of systemic inflammation [30]. The role of Gp-acetyls is somewhat unclear, but it is suggested to have pro-inflammatory properties [31]. Gp-acetyls are shown to increase with advancing age [32], and have been associated with the development of different diseases [33, 34], and shown to be a strong predictor of mortality risk [27]. Cathepsin S has previously been associated with several diseases, such as cancer, cardiovascular disease, autoimmune diseases, and pain [35], as well as total mortality, and cardiovascular and cancer mortality in older adults [28]. Higher levels of cathepsin S have shown to be associated with pro-inflammatory markers [36], as well as higher levels of CRP and IL-6 [37]. Furthermore, longitudinal studies have shown that chronic inflammation is associated with frailty, especially in women [9, 38]. In midlife, stable low levels of CRP were linked to lower odds of frailty in later life [39]. The associations between inflammatory markers and frailty vary depending on study design, frailty definitions, and included deficits. Cystatin C has also been associated with frailty, but further research is needed to establish their specific roles [40, 41].

Overall, these findings emphasize the strong connection between inflammation and frailty, suggesting that inflammatory markers could serve as important indicators and targets for interventions in frailty management.

Ageing is associated with persistent inflammation and are often affected by several co-morbidities thus requiring multiple medications. Persistent inflammation may lead to peripheral nerve sensitization causing persistent pain. Pain is often undiagnosed, but have previously been associated with increased risk of depression [42], thus there may be a link between the chronic inflammation in ageing and depression, through pain. The rates of prescriptions of antidepressant medications has raised in the past 30 years, where the largest rise of prescription is reported in older adults, mainly due to depression [43]. The high consumption of antidepressant medications is also reflected in our study population (data not shown).

Previous studies have shown a higher number of monocytes and monocyte-related genes in frail compared to non-frail subjects [10, 44, 45]. The expansion of the myeloid cell lineage is suggested to be induced by chronic low-grade inflammation [10, 45]. The dysregulation of the immune system increases the susceptibility to infections in frail subjects, and may also play a role in the pathogenesis of frailty [46]. In the present study, the gene analyses were only conducted in a female subgroup, however, previous research has revealed sex differences in the association between immune cellular profiling and frailty [47]. Still, the research on frailty and monocyte-specific gene expression is limited, and our results support the association between frailty and monocytes.

### Strengths and limitations

The main strength of the present study is the size of the study population in combination with all the measurements conducted at the study visit, which allowed us to establish a robust, retrospective FI that covered a range of systems. Most of the studies on frailty and inflammatory markers are using Fried`s definition of frailty phenotype [48], while the frailty index used is a more complex and holistic measure tool to evaluate frailty [2]. To the best of our knowledge, our study is the first to investigate the association between frailty and Gp-acetyls and cathepsin S.

Even though our FI had a balanced distribution of different deficits, and was calculated in agreement with the standard procedure by Searle et al. [2], it has not been validated. This can be a limitation in our study and may affect the comparison to studies using another FI. Also, the two commonly used definitions of frailty are very different and should rather be used as complementary frailty tools than substitutions for each other [49]. Our findings of an association between inflammation and frailty measured as FI as well as an association between frailty and monocyte-specific gene expression is supported by previous findings of an association between CRP and IL-6 with frailty using both Fried’s [25, 26, 38, 39] and FI definition [9].

The cross-sectional study design is not suitable to determine causation, and longitudinal studies are needed to determine if increased levels of inflammatory markers are caused by frailty, or if they can be used as predictors or moderators of frailty. Our study population is old and used a lot of medications that affect our physical measurements (blood pressure), and blood markers (cholesterol, inflammation). Another limitation is that the study population might not be representing the general population, due to voluntary participation, and selection bias. Also, some of the variables in the FI were based on self-reported data, which can be influenced by recall bias.

## Conclusion

Our study supports the notion that elevated levels of inflammatory markers are associated with accelerated ageing and age-related diseases and conditions, which can increase the risk of frailty. Our study showed a significant association between FI score and inflammatory markers, and FI score and monocyte-specific gene expression among elderly subjects above 70 years of age. Our study is, as far as we know, the first to show association between Gp-acetyls and frailty, and between cathepsin S and frailty. However, whether inflammation is a cause or consequence of frailty and whether the progression of frailty can be attenuated by diet and other lifestyle factors remains to be clarified.

### Electronic supplementary material

Below is the link to the electronic supplementary material.

Supplementary Material 1

## Data Availability

The datasets generated and/or analysed during the current study are available in the Gene Expression Omnibus (GEO) repository, GEO Accession Number GSE236927.

## Abbreviations

BMI: Body mass index
CVD: Cardiovascular disease
CRP: C-reactive protein
FI: Frailty Index
Gp-acetyls: Glycoprotein- acetyls
HbA1c: Hemoglobin A1c
HDL-C: High density lipoprotein- cholesterol
IGF-1: Insulin-like growth factor 1
IL-6: Interleukin-6
LDL-C: Low Density Lipoprotein- Cholesterol
MMSE: Mini Mental Status Evaluation
PBMC: Peripheral blood mononuclear cell
SPPB: Short Physical Performance Battery
TC: Total Cholesterol
TG: Triglycerides
