# Supplementary material for: Inflammatory markers and frailty in home-dwelling elderly, a cross-sectional study
Source: BMC Geriatr. 2024 Feb 19;24:175. doi: 10.1186/s12877-024-04690-2 (PMC10877806; doi:10.1186/s12877-024-04690-2)
Supplement: Supplementary file 1 — Supplementary Material 1 [file 12877_2024_4690_MOESM1_ESM.docx]

**Supplementary Table 1**. Descriptive data in frail and non-frail women and men

| Variable | Frail women (N=40) | Frail men  (N=30) | P-value^1^ | Non-frail women  (N=170) | Non-Frail men  (N=163) | P-value^2^ | P-value^3^ | P-value^4^ |
| --- | --- | --- | --- | --- | --- | --- | --- | --- |
| Age, years | 78 (70-90) | 78 (70-90) | 0.73 | 74 (70-91) | 74(70-93) | 0.73 | **<0.001** | **<0.01** |
| BMI, kg/m^2^ | 26.9 ± 4.3 | 26.7 ± 4.7 | 0.89 | 26.0 ± 4.3 | 26.1 ± 3.2 | 0.93 | 0.25 | 0.32 |
| Daily smoking, n (%) | 1 (3) | 2 (7) | 0.57 | 12 (7) | 9 (6) | 0.56 | 0.47 | 0.68 |
| Living alone, n (%) | 22 (55) | 12 (40) | 0.21 | 74 (44) | 29 (18) | **<0.001** | 0.19 | **<0.01** |
| FI score | 0.31 (0.25-0.47) | 0.31(0.25-0.56) | 0.56 | 0.14 ± 0.06 | 0.13 ± 0.06 | 0.09 | **<0.001** | **<0.001** |
| *Blood markers* |  |  |  |  |  |  |  |  |
| Triglycerides, mmol/l | 1.24 (0.5-3.5) | 1.21 (0.68-4.1) | 0.54 | 1.38(0.59-4.89) | 1.3 (0.5-5.04) | 0.64 | 0.57 | 0.39 |
| Total Cholesterol, mmol/l | 5.4 ± 1.0 | 4.6 ± 1.1 | **0.002** | 5.7 ± 1.0 | 5.0 ± 1.1 | **<0.001** | 0.13 | 0.06 |
| LDL-cholesterol, mmol/l | 3.1 ± 1.0 | 2.6 ± 1.0 | ***0.07*** | 3.3 ± 0.9 | 3.0 ± 1.0 | **<0.01** | 0.12 | ***0.05*** |
| HDL-cholesterol, mmol/l | 1.8 (0.7-3) | 1.3 (0.7-3.6) | **<0.001** | 1.8 ± 0.5 | 1.4 ± 0.4 | **<0.001** | 0.72 | 0.61 |
| HbA1c, mmol/mol | 6.0 ± 0.5 | 5.8 ± 0.6 | 0.28 | 6.0 ± 0.7 | 5.9 ± 0.7 | 0.41 | 0.97 | 0.61 |
| Glucose, mmol/l | 4.4 (3.6-9.7) | 4.3 (3.2-7.1) | 0.52 | 4.4 (3.0-20.7) | 4.5 (3.0-15.5) | 0.08 | 0.56 | 0.27 |

*Cut-off value to be categorized as “frail” was set to ≥0.25. Parametric data are presented as mean ±SD, non-parametric data are presented as median (min-max). P-values: Continuous parametric data were tested by t-test, continuous non-parametric data were tested by Mann- Whitney U test, Chi-square test was used for categorical data, while Fisher's exact test was used for small groups. Statistical significant level: P-value <0.05. BMI, body mass index; FI-score, Frailty Index score; LDL, low-density lipoprotein; HDL, high-density lipoprotein. P-value^1^; Frail women vs frail men, P-value^2^; Non-frail women vs non-frail men, P-value^3^; Frail women vs Non-frail women, P-value^4^; Frail men vs Non-frail men*

**Supplementary Table 2a.** The 38 variables/deficits included in the frailty index

|  | Deficit | Code |
| --- | --- | --- |
| 1 | **Cancer** | Yes=1/no=0 |
| 2 | **Inflammatory disease** | Yes=1/no=0 |
| 3 | **Pulmonary disease** | Yes=1/no=0 |
| 4 | **Diabetes** | Yes=1/no=0 |
| 5 | **Use of TSH medications** | Yes=1/no=0 |
| 6 | **Cardiovascular disease** | Yes=1/no=0 |
| 7 | **Blood pressure-lowering medications** | Yes=1/no=0 |
| 8 | **Low blood levels of hemoglobin** | Yes=1/no=0 |
| 9 | **BMI outside of range (<22- ≤27)** | Yes=1/no=0 |
| 10 | **Polypharmacy  (≥5 daily)** | Yes=1/no=0 |
| 11 | **Moderate activities** | Major limitations =1/some limitations =0,5/ no limitations =0 |
| 12 | **Lift/carry a shopping basket** | Major limitations =1/some limitations =0,5/ no limitations =0 |
| 13 | **Mobility** | bedridden/sitting at a chair=1, can stand up from bed/chair, but can't walk alone = 0,5/can walk outside =0 |
| 14 | **Food intake** | Not able to eat by itselfes=1/ can eat by itself but with some limitations =0,5/ eat by itself with no limitations =0 |
| 15 | **Walk stairs, one floor** | Major limitations =1/some limitations =0,5/ no limitations =0 |
| 16 | **Bend or squat** | Major limitations =1/some limitations =0,5/ no limitations =0 |
| 17 | **Walk a few hundred meters** | Major limitations =1/some limitations =0,5/ no limitations =0 |
| 18 | **Wash or dress yourself** | Major limitations =1/some limitations =0,5/ no limitations =0 |
| 19 | **SPPB: chair test** | 0p on the test =1/1p on the test = 0,75/ 2 p on the test =0,5 / 3p on the test = 0,25/ 4p on the test =0 |
| 20 | **SPPB: walking speed** | 0p on the test =1/1p on the test = 0,75/ 2 p on the test =0,5 / 3p on the test = 0,25/ 4p on the test =0 |
| 21 | **SPPB: balance test** | 0p on the test =1/1p on the test = 0,75/ 2 p on the test =0,5 / 3p on the test = 0,25/ 4p on the test =0 |
| 22 | **Grip strength (Dominant, kg)** | Men: BMI ≤ 24, GS ≤ 29, BMI 24.1-28, GS ≤ 30, BMI >28, GS ≤ 32 Women: BMI ≤ 23, GS ≤ 17, BMI23.1-26, GS≤ 17.3, BMI 26.1-29, GS≤ 18, BMI >29, GS≤ 21 = 1, if higher = 0 |
| 23 | **Weight loss last 3 months** | >3kg weight loss =1/ 1-3kg weight loss or "don't know" = 0,5/  no weight loss = 0 |
| 24 | **My health is …** | Poor=1/ quite good =0,75/ good= 0,5/very good =0,25/ excellent =0 |
| 25 | **My health today compared with one year ago is…** | 1= much worse/ 0,5= a little worse/ the same or a little better or much better =0 |
| 26 | **I'm just as healthy as most people I know*** | completely wrong=1/ partial wrong=0,75/don't know=0,5/ partial right=0,25/completely right=0 |
| 27 | **Been so far down mentally that no one can cheer me up** | All the time =1 /much of the time=0,75/part of the time =0,5/ a little of the time=0,25/not at all=0 |
| 28 | **Felt depressed*** | All the time =1 /much of the time=0,75/part of the time =0,5/ a little of the time=0,25/not at all=0 |
| 29 | **Physical or emotional problems have obstructed social interactions*** | All the time =1 /much of the time=0,75/part of the time =0,5/ a little of the time=0,25/not at all=0 |
| 30 | **Felt happy*** | Not at all=1/ a little of the time=0,75/ part of the time=0,5/  much of the time=0,25/ all of the time=0 |
| 31 | **Felt filled with life *** | Not at all=1/ a little of the time=0,75/ part of the time=0,5/  much of the time=0,25/ all of the time=0 |
| 32 | **Had a lot of energy*** | Not at all=1/ a little of the time=0,75/ part of the time=0,5/  much of the time=0,25/ all of the time=0 |
| 33 | **Felt tired *** | All the time =1 /much of the time=0,75/part of the time =0,5/ a little of the time=0,25/not at all=0 |
| 34 | **Felt fatigue *** | All the time =1 /much of the time=0,75/part of the time =0,5/ a little of the time=0,25/not at all=0 |
| 35 | **Time orientation (MMSE)** | Two or more answers wrong=1 / one answer wrong=0,5/  Five answers right=0 |
| 36 | **Place orientation (MMSE)** | Two or more answers wrong=1 / one answer wrong=0,5/  Five answers right=0 |
| 37 | **Attention and mental calculations (MMSE)** | Two or more answers wrong=1 / one answer wrong=0,5/  Five answers right=0 |
| 38 | **Deferred recall (MMSE)** | One or zero answer right=1/ two answer wrong=0,5/  Three answers right=0 |

**Last 4 weeks
SPPB; Short Physical Performance battery (49), MMSE; Mini Mental Status Evaluation (50)*

**Supplementary Table 2b.** Distribution of frailty variables in men and women in general and in frail women and men

| Variable | N total | Women (N=210) | Men (N=193) | P-value_1_ | Frail Women (N=40) | Frail Men (N=30) | P-value_2_ |
| --- | --- | --- | --- | --- | --- | --- | --- |
| Cancer, n (%) | 403 | 20 (10) | 37 (19) | **0.006** | 6 (15) | 12 (40) | **0.02** |
| Inflammatory disease, n (%) | 403 | 56 (27) | 42 (22) | 0.25 | 20 (67) | 10 (33) | 0.16 |
| Pulmonary disease, n (%) | 403 | 21 (10) | 18 (9) | 0.82 | 8 (20) | 9 (30) | 0.33 |
| Diabetes, n (%) | 403 | 11 (5) | 8 (4) | 0.61 | 3 (8) | 0 (0) | 0.26 |
| Use of TSH medications, n (%) | 403 | 22 (11) | 6 (3) | **0.004** | 7 (18) | 1 (3) | 0.13 |
| Cardiovascular disease, n (%) | 403 | 43 (21) | 71 (37) | **<0.001** | 18 (45) | 18 (60) | 0.21 |
| Blood pressure lowering medications, n (%) | 401 | 98 (47) | 88 (46) | 0.91 | 25 (63) | 21 (70) | 0.51 |
| Low blood levels of hemoglobin, n (%) | 402 | 6 (3) | 17 (9) | **0.01** | 3 (8) | 7 (23) | ***0.09*** |
| BMI outside of range (<22- ≤27), n (%) | 403 | 108 (51) | 83 (43) | ***0.09*** | 23 (58) | 18 (60) | 0.83 |
| Polypharmacy (≥5 daily), n (%) | 401 | 23 (11) | 38 (20) | **0.01** | 11 (28) | 21 (70) | **<0.001** |
| Moderate activities, n (%) | 401 |  |  | **0.001** |  |  | 0.18 |
| *some limitations* |  | 54 (26) | 33 (17) |  | 23 (58) | 16 (55) |  |
| *major limitations* |  | 19 (9) | 6 (3) |  | 13 (33) | 6 (21) |  |
| Lift/carry shopping basket, n (%) | 400 |  |  | **<0.001** |  |  | **0.003** |
| *some limitations* |  | 50 (24) | 21 (11) |  | 25 (63) | 11 (38) |  |
| *major limitations* |  | 14 (7) | 5 (3) |  | 10 (25) | 5 (17) |  |
| Mobility, n (%) | 403 |  |  | 0.43 |  |  | 0.65 |
| *some limitations* |  | 2 (1) | 4 (2) |  | 2 (5) | 3 (10) |  |
| *major limitations* |  | 0 (0.) | 0 (0) |  | 0 (0) | 0 (0) |  |
| Food intake, n (%) | 403 |  |  | 0.48 |  |  | 0.43 |
| *some limitations* |  | 0 (0) | 1 (1) |  | 0 (0) | 1 (3) |  |
| *major limitations* |  | 0 (0) | 0 (0) |  | 0 (0) | 0 (0) |  |
| Walk stairs, one floor, n (%) | 398 |  |  | 0.10 |  |  | 0.62 |
| *some limitations* |  | 23 (11) | 15 (8) |  | 16 (40) | 11 (38) |  |
| *major limitations* |  | 9 (4) | 4 (2) |  | 8 (20) | 4 (14) |  |
| Bend or squat, n (%) | 401 |  |  | ***0.05*** |  |  | 0.74 |
| *some limitations* |  | 76 (36) | 62 (32) |  | 19 (48) | 15 (52) |  |
| *major limitations* |  | 22 (11) | 9 (5) |  | 14 (35) | 8 (28) |  |
| Walk a few hundred meters,  n (%) | 400 |  |  | 0.20 |  |  | 0.93 |
| *some limitations* |  | 25 (12) | 19 (10) |  | 15 (38) | 12 (41) |  |
| *major limitations* |  | 12 (6) | 6 (3) |  | 8 (20) | 5 (17) |  |
| Wash or dress yourself, n (%) | 401 |  |  | 0.84 |  |  | 0.11 |
| *some limitations* |  | 10 (5) | 11 (6) |  | 6 (15) | 9 (31) |  |
| *major limitations* |  | 3 (1) | 2 (1) |  | 2 (5) | 2 (7) |  |
| SPPB: chair test, n (%) | 403 |  |  | 0.32 |  |  | 0.79 |
| *4p* |  | 112 (53) | 113 (59) |  | 10 (25) | 9 (30) |  |
| *3p* |  | 58 (28) | 55 (29) |  | 5 (13) | 9 (30) |  |
| *2p* |  | 23 (11.) | 19 (10) |  | 12 (30) | 7 (23) |  |
| *1p* |  | 10 (5) | 5 (3) |  | 7 (18) | 4 (13) |  |
| *0p* |  | 7 (3) | 1 (1) |  | 6 (15) | 1 (3) |  |
| SPPB: walking speed, n (%) | 403 |  |  | 0.30 |  |  | 1.0 |
| *4p* |  | 200 (95) | 188 (97) |  | 33 (83) | 25 (83) |  |
| *3p* |  | 6 (3) | 2 (1) |  | 5 (13) | 2 (7) |  |
| *2p* |  | 2 (1) | 2 (1) |  | 1 (3) | 2 (7) |  |
| *1p* |  | 0 (0) | 1 (1) |  | 0 (0) | 1 (3) |  |
| *0p* |  | 2 (1) | 0 (0) |  | 1 (3) | 0 (0) |  |
| SPPB: balance test, n (%) | 403 |  |  | ***0.07*** |  |  | 0.60 |
| *4p* |  | 173 (82) | 172 (89) |  | 26 (65) | 22 (73) |  |
| *3p* |  | 20 (10) | 11 (6) |  | 5 (13) | 4 (13) |  |
| *2p* |  | 9 (4) | 6 (3) |  | 6 (15) | 0 (0) |  |
| *1p* |  | 5 (2) | 2 (1) |  | 2 (5) | 2 (7) |  |
| *0p* |  | 3 (1) | 2 (1) |  | 1 (3) | 2 (7) |  |
| Low grip strength, n (%) | 402 | 42 (20) | 21 (11) | **0.01** | 17 (43) | 11 (37) | 0.62 |
| Weight loss last 3 months, n (%) | 403 |  |  | 1.00 |  |  | 1.0 |
| *1-3kg or "don`t know"* |  | 26 (12) | 20 (10) |  | 10 (25) | 5 (17) |  |
| *>3kg* |  | 0 (0) | 4 (2) |  | 0 (0) | 2 (7) |  |
| My health is…, n (%) | 402 |  |  | 0.71 |  |  | 0.50 |
| *Excellent* |  | 18 (8) | 14 (7) |  | 2 (5) | 0 (0) |  |
| *very good* |  | 74 (35) | 73 (38) |  | 3 (8) | 5 (17) |  |
| *good* |  | 93 (45) | 86 (45) |  | 19 (48) | 10 (33) |  |
| *Quite good* |  | 22 (11) | 17 (9) |  | 14 (35) | 12 (40) |  |
| *Poor* |  | 2 (1.0) | 3 (1.6) |  | 2 (5.0) | 3 (10.0) |  |
| My health today compared with a year ago is…, n (%) | 402 |  |  | 0.66 |  |  |  |
| *The same/a little better/ much better* |  | 183 (88) | 166 (86) |  | 22 (55) | 20 (67) | 0.46 |
| *A little worse* |  | 24 (12) | 25 (12) |  | 16 (40) | 9 (30) |  |
| *Much worse* |  | 2 (1) | 2 (1) |  | 2 (5) | 1 (3) |  |
| I`m just as healthy as most people I know, n (%) | 402 |  |  | 1.0 |  |  | 0.77 |
| *Completely right* |  | 127 (61) | 117 (61) |  | 8 (20) | 5 (17) |  |
| *Partial right* |  | 45 (22) | 32 (17) |  | 14 (35) | 8 (27) |  |
| *Don`t know* |  | 22 (11) | 23 (12) |  | 12 (30) | 7 (23) |  |
| *Partial wrong* |  | 4 (2) | 8 (4) |  | 4 (10) | 6 (20) |  |
| *Completely wrong* |  | 11 (5) | 13 (7) |  | 2 (5) | 4 (13) |  |
| Been so far down mentally that no one can cheer me up*, n (%) | 401 |  |  | 0.39 |  |  | 0.81 |
| *Not at all* |  | 175 (84) | 269 (88) |  | 23 (58) | 19 (63) |  |
| *A little of the time* |  | 29 (14) | 19 (10) |  | 14 (35) | 7 (23) |  |
| *Part of the time* |  | 1 (1) | 4 (3) |  | 0 (0) | 3 (10) |  |
| *Much of the time* |  | 3 (1) | 1 (1) |  | 3 (8) | 1 (3) |  |
| *All the time* |  | 0 (0) | (0) |  |  |  |  |
| Felt depressed*, n (%) | 400 |  |  | 0.15 |  |  | 0.81 |
| *Not at all* |  | 144 (69) | 146 (76) |  | 17 (44) | 14 (47) |  |
| *a little of the time* |  | 52 (25) | 34 (18) |  | 16 (41) | 7 (23) |  |
| *part of the time* |  | 9 (4) | 9 (5) |  | 4 (10) | 6 (20) |  |
| *much of the time* |  | 2 (1) | 2 (1) |  | 1 (3) | 2 (7) |  |
| *All the time* |  | 1 (1) | 1 (1) |  | 1 (3) | 1 (3) |  |
| Physical or emotional problems have obstructed social interactions*,  n (%) | 400 |  |  | 0.50 |  |  | 0.59 |
| *Not at all* |  | 147 (78) | 142 (74) |  | 10 (26) | 10 (35) |  |
| *a little of the time* |  | 40 (19) | 30 (16) |  | 16 (41) | 8 (28) |  |
| *part of the time* |  | 16 (8) | 15 (8) |  | 10 (26) | 6 (21) |  |
| *much of the time* |  | 4 (2) | 3 (2) |  | 3 (8) | 3 (10) |  |
| *All the time* |  | 1 (1) | 2 (1) |  | 0 (0) | 2 (7) |  |
| Felt happy*, n (%) | 402 |  |  | 0.70 |  |  | 0.28 |
| *All of the time* |  | 37 (18) | 37 (19) |  | 4 (10) | 1 (3) |  |
| *much of the time* |  | 110 (53) | 100 (52) |  | 10 (25) | 7 (23) |  |
| *part of the time* |  | 35 (17) | 30 (16) |  | 14 (35) | 11 (37) |  |
| *a little of the time* |  | 21 (10) | 16 (8) |  | 10 (25) | 7 (23) |  |
| *not at all* |  | 6 (3) | 10 (5) |  | 2 (5) | 4 (13) |  |
| Felt filled with life*, n (%) | 400 |  |  | 0.06 |  |  | 0.57 |
| *All of the time* |  | 24 (12) | 35 (18) |  | 1 (3) | 2 (7) |  |
| *much of the time* |  | 79 (38) | 85 (44) |  | 3 (8) | 3 (10) |  |
| *part of the time* |  | 69 (33) | 44 (23) |  | 18 (45) | 8 (28) |  |
| *a little of the time* |  | 27 (13) | 23 (12.) |  | 11 (28) | 12 (41) |  |
| *not at all* |  | 9 (4) | 5 (3) |  | 7 (18) | 4 (14) |  |
| Had a lot of energy*, n (%) | 402 |  |  | **0.004** |  |  | 0.18 |
| *All of the time* |  | 17 (8) | 34 (18) |  | 0 (0) | 2 (7) |  |
| *much of the time* |  | 85 (41) | 78 (40) |  | 4 (10) | 2 (7) |  |
| *part of the time* |  | 66 (32) | 48 (25) |  | 17 (43) | 8 (27) |  |
| *a little of the time* |  | 28 (13) | 21 (11) |  | 12 (30.0) | 10 (33) |  |
| *not at all* |  | 13 (6) | 25 (6) |  | 7 (18) | 8 (27) |  |
| Felt tired*, n (%) | 401 |  |  | 0.41 |  |  | 1.0 |
| *Not at all* |  | 45 (22) | 49 (25) |  | 4 (10) | 3 (10) |  |
| *a little of the time* |  | 107 (51) | 96 (50) |  | 10 (25) | 10 (33) |  |
| *part of the time* |  | 39 (19) | 36 (19) |  | 16 (40) | 9 (30) |  |
| *much of the time* |  | 16 (8) | 10 (5) |  | 9 (23) | 6 (20) |  |
| *All the time* |  | 1 (1) | 2 (1) |  | 1 (3) | 2 (7) |  |
| Felt fatigue*, n (%) | 402 |  |  | ***0.054*** |  |  | 0.69 |
| *Not at all* |  | 38 (18) | 51 (26) |  | 4 (10) | 2 (7) |  |
| *a little of the time* |  | 111 (53) | 100 (52) |  | 16 (40) | 11 (37) |  |
| *part of the time* |  | 49 (23) | 34 (18) |  | 13 (33) | 9 (30) |  |
| *much of the time* |  | 9 (4) | 5 (3) |  | 5 (13) | 5 (17) |  |
| *All the time* |  | 2 (1) | 3 (2) |  | 2 (5) | 3 (10) |  |
| *MMSE: Time orientation,* n (%) | **401** |  |  | 0.66 |  |  | 1.0 |
| *5 answer right* |  | 183 (88) | 166 (86) |  | 30 (75) | 22 (73) |  |
| *1 answer wrong* |  | 21 (10) | 20 (10) |  | 8 (20) | 3 (10) |  |
| *≥2 answers wrong* |  | 4 (2) | 7 (4) |  | 2 (5) | 5 (17) |  |
| MMSE: Place orientation, n (%) | 401 |  |  | 0.45 |  |  | 0.60 |
| *5 answer right* |  | 185 (89) | 166 (86) |  | 26 (65) | 22 (73) |  |
| *1 answer wrong* |  | 19 (9) | 23 (12) |  | 11 (28) | 6 (20) |  |
| *≥2 answers wrong* |  | 4 (2) | 4 (2) |  | 3 (8) | 2 (7) |  |
| MMSE: Attention and mental calculations, n (%) | 401 |  |  | ***0.07*** |  |  | 0.88 |
| *5 answer right* |  | 102 (49) | 112 (58) |  | 14 (35) | 10 (33) |  |
| *1 answer wrong* |  | 9 (4) | 6 (3) |  | 3 (8) | 0 (0) |  |
| *≥2 answers wrong* |  | 97 (47) | 75 (39) |  | 23 (58) | 20 (67) |  |
| Deferred recall (MMSE), n (%) | 401 |  |  | 0.22 |  |  | 0.30 |
| *≤ 1 answer right* |  | 162 (78) | 140 (73) |  | 25 (63) | 15 (50) |  |
| *2 answer wrong* |  | 36 (17) | 40 (21) |  | 8 (20) | 9 (30) |  |
| *3 answers right* |  | 10 (5) | 13 (7) |  | 7 (18) | 6 (20) |  |

**Last 4 weeks*
*SPPB; Short Physical Performance battery (59), MMSE; Mini Mental Status Evaluation (60)*

**Supplementary Table 3a.** Inflammatory blood markers in the study population

| Inflammatory markers | Total (N=403) | Frail (N=70) | Non-frail (N=333) | P-value |
| --- | --- | --- | --- | --- |
| IL-6, pg/mL | 1.41 (0.37-10.0) | 1.77 (0.47-8.90) | 1.33 (0.37-10.0) | **<0.001** |
| CRP*, mg/L | 1.5 (0.1-23) | 1.8 (0.3-17) | 1.5 (0.1-23) | 0.13 |
| IGF-1, ng/mL | 87.14 ± 25.82 | 88.65 ± 29.24 | 86.82 ± 25.08 | 0.59 |
| Cystatin C, ng/mL | 792.07 (496.87-1871.1) | 852.9 (551.2-1864.6) | 778.9 (496.9-1871.1) | **<0.001** |
| Cathepsin S, pg/mL | 8051.04 ± 2020.51 | 8384.8 ± 2114.0 | 7980.9 ± 1996.5 | **0.01** |
| Gp-acetyls, mmol/L | 1.27 ± 0.20 | 1.33 ± 0.26 | 1.26 ± 0.18 | **0.01** |

*Parametric data are presented as mean ±SD, non-parametric data are presented as median (min-max). P-values: Continuous parametric data were tested by t-test, continuous non-parametric data were tested by Mann-Whitney U test, * CRP levels ≥ 50 were excluded from the analysis*. *IL-6; interleukin-6, IGF-1; insulin-like growth factor-1, CRP; C-reactive protein, Gp-acetyls;* Glycoprotein acetyls.

**Supplementary Table 3b.** Inflammatory blood markers in frail and non-frail women and men

| Inflammatory markers | Frail women (N=40) | Frail men  (N=30) | P-value^1^ | Non-Frail women  (N=170) | Non-Frail men  (N=163) | P-value^2^ | P-value^3^ | P-value^4^ |
| --- | --- | --- | --- | --- | --- | --- | --- | --- |
| IL-6, pg/mL | 1.63 (0.47-5.85) | 2.00 (0.70-8.90) | 0.40 | 1.3 (0.45-10.0) | 1.4 (0.37-8.16) | 0.10 | **0.01** | **0.01** |
| CRP*, mg/L | 1.6 (0.3-13) | 2.5 (0.4-17) | 0.27 | 1.6 (0.1-14) | 1.3 (0.2-23) | 0.18 | 0.95 | ***0.05*** |
| IGF-1, ng/mL | 87.82 ± 30.17 | 89.74 ± 28.43 | 0.79 | 84.0 ± 23.39 | 89.76 ± 26.47 | **0.04** | 0.38 | 0.997 |
| Cystatin C, ng/mL | 827.5  (551.3-1435.5) | 931.9  (664.4-1864.6) | **0.03** | 764.7  (538.1-1871.1) | 806.6  (496.9-1403.4) | **<0.01** | **0.02** | **<0.01** |
| Cathepsin S, pg/mL | 8474.5 ± 2029.6 | 8265.3 ± 2251.1 | 0.69 | 7846.2 ± 1889.1 | 8121.3 ± 2099.3 | 0.21 | ***0.06*** | 0.73 |
| Gp-acetyls, mmol/L | 1.31 (1.02-1.9) | 1.24 (0.89-2.6) | 0.36 | 1.27 ± 0.18 | 1.24 ± 0.19 | 0.17 | ***0.09*** | 0.33 |

*Cut-off value to be categorized as “frail” was set to ≥0.25. Parametric data are presented as mean ±SD, non-parametric data are presented as median (min-max). P-values: Continuous parametric data were tested by t-test, continuous non-parametric data were tested by Mann- Whitney U test. *CRP: <50, outliers are removed from the analyses, N=400. P-value^1^; Frail women vs frail men, P-value^2^; Non-frail women vs non-frail men, P-value^3^; Frail women vs Non-frail women, P-value^4^; Frail men vs Non-frail men. IL-6; interleukin-6, IGF-1; insulin-like growth factor-1, CRP; C-reactive protein, Gp-acetyls;* Glycoprotein acetyl

**Supplementary Table 4.** Inflammatory blood markers and frailty index score in multiple linear regression model.

| Inflammatory marker | N | Β- coefficient† | 95% CI † | P-value † | Β-coefficient^ɸ^ | 95% CI^ɸ^ | P-value ^ɸ^ |
| --- | --- | --- | --- | --- | --- | --- | --- |
| IL6^^^, % | 394 | 0.002 | 0.001, 0.002 | **<0.001** | 0.002 | 0.001, 0.002 | **<0.001** |
| CRP^*^^, % | 400 | 0.0009 | 0.0004, 0.0012 | **<0.001** | 0.0003 | (-0.0001), 0.0007 | 0.13 |
| IGF-1, ng/ml | 403 | -0.0002 | (-0.0006), 0.0002 | 0.276 | (-1.27e-06) | (-0.0003), 0.0003 | 0.99 |
| Cystatin C^^^, % | 403 | 0.006 | 0.004, 0.008 | **<0.001** | 0.004 | 0.002, 0.006 | **<0.001** |
| Cathepsin S, pg/ml | 403 | 8.59e-06 | 3.95e-06, 0.00001 | **<0.001** | 6.7e-06 | 2.44e-06, 0.00001 | **0.002** |
| Gp-acetyls, mmol/L | 403 | 0.095 | 0.05, 0.14 | **<0.001** | 0.09 | 0.05, 0.13 | **<0.001** |

*† Unadjusted, ^ɸ^ Adjusted for age, sex, BMI, and smoking, ^Log-transformed,* CRP<50;* *CRP levels ≥ 50 were excluded from the analysis, 95% CI; 95% confidence interval, IL-6; interleukin-6, IGF-1; insulin-like growth factor-1, CRP; C-reactive protein,* Gp-acetyls*;* Glycoprotein acetyls*. Beta-coefficients and 95% CIs are multiplied with 0,1 to describe the change in inflammatory marker for each 0,1 unit change in FI score.*

**Supplementary Table 5:** Descriptive data of the sub-group with gene expression available

| Variable | Total (N=89) | Frail (N=19) | Non-frail (N=70) | P-value |
| --- | --- | --- | --- | --- |
| Age, years | 78.1 ± 5.2 | 80.0 ± 3.6 | 76 (70-93) | **0.02** |
| Female, n (%) | 100 | 100 | 100 |  |
| BMI, kg/m^2^ | 25.5 ± 4.1 | 26.5 ± 3.5 | 25.2 ± 4.2 | 0.25 |
| Daily smoking, n (%) | 0 (0) | 0 (0) | 0 (0) |  |
| Single/living alone, n (%) | 47 (53) | 14 (74) | 33 (47) | **0.04** |
| FI score | 0.19 ± 0.1 | 0.33 ± 0.06 | 0.15 ± 0.06 | **<0.001** |

*Parametric data are presented as mean ± SD, non-parametric data are presented as median (min-max). P-values: Continuous parametric variables were tested by t-test- Continuous non-parametric variables were tested by Mann-Whitney U test. Categorical data were tested by chi-square test. Statistical significant level: P-value <0.05. BMI, body mass index; FI score, frailty index score.*

**Supplementary Figure 1:** The flow to establish the frailty index

**
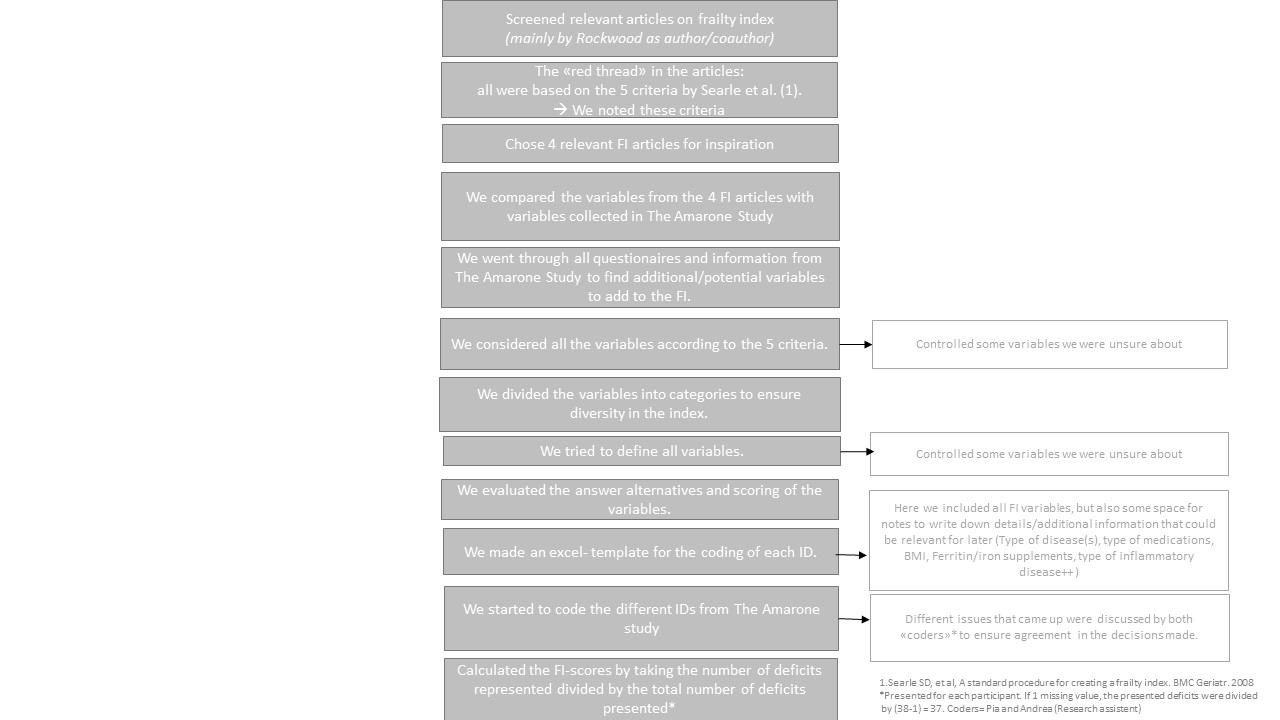
**

**Supplementary Figure 2:** Association between frailty index score and serum inflammatory markers in the subpopulation (N=89)


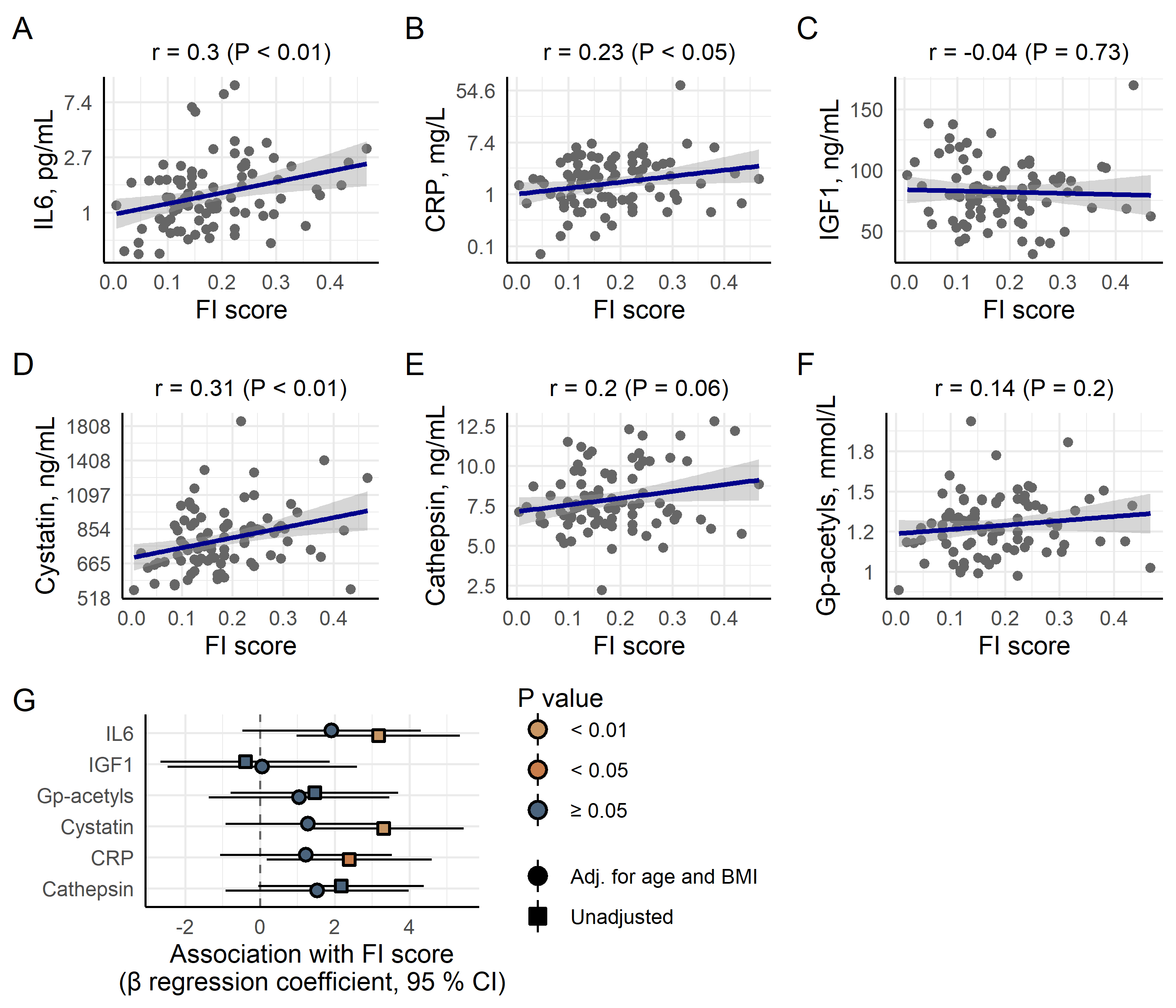


**Figure Texts:**

**Supplementary Figure 1:** The flow describes the process to construct our frailty index, retrospectively. We used the procedure by Searle et al.(2) which resulted in 38 deficits.

**Supplementary Figure 2:** The scatter plots (panels A-F) show the association between FI score and inflammatory markers in the subpopulation (N=89), along with a linear regression trend line; Spearman’s correlation coefficients are displayed in headers, with corresponding P values. Note that IL-6, CRP, Cystatin, and Gp-acetyls were log-transformed, while the axis labels were back-transformed. The forest plot (panel G) shows the unadjusted and adjusted (for age and BMI) β regression coefficients with 95 % confidence intervals (CIs), colored by P value. Abbreviations: BMI, body mass index; CRP, C-reactive protein; FI, frailty index; IGF1, insulin-like growth factor-1; IL-6, interleukin 6; Gp-acetyls, glycoprotein acetyls.
